# Multiple Pigmented Bowen's Disease: A Diagnostic and Therapeutic Dilemma

**DOI:** 10.1155/2012/342030

**Published:** 2012-10-16

**Authors:** Pratik Gahalaut, Madhur Kant Rastogi, Nitin Mishra, Sandhya Chauhan

**Affiliations:** ^1^Department of Dermatology, Shri Rammurti Smarak Institute of Medical Sciences, Nainital Road, Bareilly 243006, India; ^2^Pediatric Oncology Unit, Department of Pediatrics, Shri Rammurti Smarak Institute of Medical Sciences, Nainital Road, Bareilly 243006, India

## Abstract

Bowens' disease (BD) is a precancerous condition of skin and/or mucosa with a predilection towards sun-exposed areas. Extensive literature research failed to reveal any case of multiple pigmented BD in type V Fitzpatrick skin. Multiple BD is a therapeutic challenge with a tendency to recur. Here we present an otherwise healthy Indian male having multiple pigmented lesions of BD on sun-protected sites of the body mimicking malignant melanoma. These lesions were refractory to treatment with different modalities. This paper is an attempt to review the available literature regarding the pigmented variant of multiple BD. Rationale for a therapeutic trial of UVB therapy for multiple arsenic induced BD is also discussed.

## 1. Introduction

BD is a form of intraepidermal squamous cell carcinoma in situ with a small potential of invasive malignancy [1]. It may affect both skin and/or mucosa. It usually occurs as a solitary lesion on sun-exposed areas such as the head and neck [2]. BD is uncommon in individuals with pigmented skin and multiple lesions occur in only 10–20% of patients [1, 2]. Further pigmented variant is very rare and incidence is <2% [2–4]. Though solitary pigmented lesions of BD have been reported earlier, extensive search failed to reveal any case of generalized pigmented BD on sun protected sites in type V Fitzpatrick skin. 

## 2. Case Report

**Figure 1:**
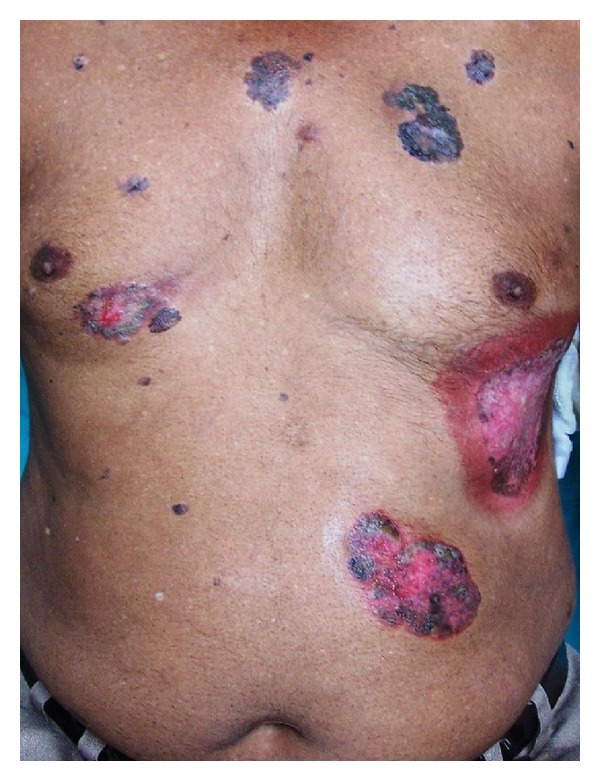
Multiple hyperpigmented plaques and erosions of irregular shapes and sizes with and without scaling on the trunk.

**Figure 2:**
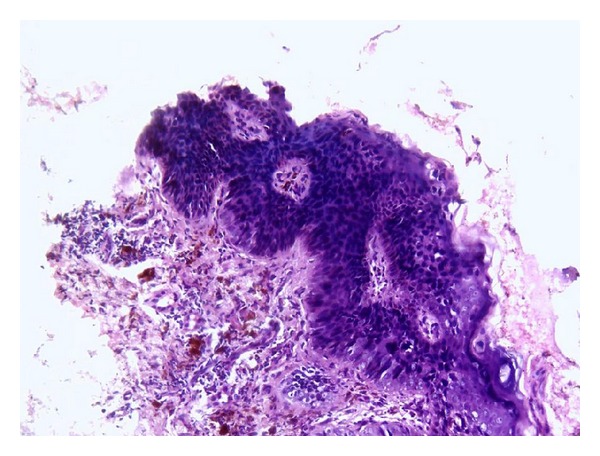
Epidermis shows thickening of rete ridges and parakeratosis along with proliferation of atypical squamous epithelial cell. Basal cell layer is sharply demarcated from underlying dermis. (HE stain, magnification 10x).

**Figure 3:**
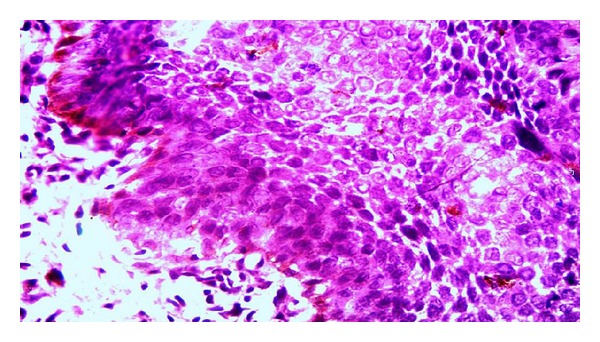
Melanin pigment seen in upper and basal epithelial cell layers. (HE stain, magnification 40x).

A 68 years old married, retired male clerk presented with slowly progressive, multiple erythematous and/or hyper pigmented, scaly, crusted irregular erosions and plaques over trunk for the last 10 years approximately. These were associated with itching and/or burning sensation intermittently. Lesions ranged from 0.5 × 0.5 cm^2^ to 10 × 10 cm^2^ approximately. Majority of lesions were present on sun-protected sites (Figure 1). Few lesions were tender. Lesions were unresponsive to various therapeutic modalities including cryotherapy, topical antifungals, retinoid, steroid, 5-fluorouracil and imiquimod applications. Patient was nonalcoholic and nonsmoker and denied any history of extramarital sexual exposure. There was no history suggestive of underlying malignancy or immunocompromised state. Family history failed to reveal any malignancy or any sign suggestive of arsenic toxicity. He gave history of long term homeopathic treatment for general well being for approximately 10 years about 20 years back which was stopped after cutaneous lesions started appearing. Systemic examination was unremarkable and vitals were stable. Mucosae were spared and there was no other clinical evident sign suggestive of chronic arsenic ingestion. Complete blood hemogram and routine urine microscopic examination was within normal limits. ELISA for HIV (human immunodeficiency virus) was negative. Serum arsenic level was 15 *μ*gm/litre (normal <60 *μ*gm/litre). Biopsy was taken from a plaque which showed disorganized proliferation of atypical squamous epithelial cells with disproportionately enlarged hyperchromatic nucleus. Few cells in upper epidermis showed vacuolation. Basal cell layer was sharply demarcated from underlying dermis (Figure 2). Melanin pigment was increased in basal epidermal cell layer (Figure 3). As our patient had tried multiple modalities in vain, he was counselled about the disease and the various treatment options available. He was started on trice weekly course of NBUVB (Narrowband UVB) therapy on alternate days, after taking an informed written consent. Baseline biopsies were taken from multiple plaques for ascertaining the response of phototherapy. Unfortunately he was lost to follow-up after three weeks. During this period there was no perceptible clinical change in his lesions. 

## 3. Discussion 

Irradiation in the form of solar, radiotherapy, or photochemotherapy; carcinogens like arsenic; immunosuppresion either therapeutic or pathological; certain types of HPV (human papilloma virus); chronic injury, and rarely pre-existing skin lesions like seborrhoeic keratosis have been implicated as etiological factors for developing BD over several years [1, 2, 5, 6]. 

Occurrence of BD in pigmented skin warrants a search for etiological factors other than irradiation [1, 7]. HPV is mainly related to the development of BD in non sun-exposed areas such as anogenital region, palms, soles, and mucosa [8]. Up to 30 to 58% of extragenital BD lesions harbour HPV DNA and have been related to infection with HPV subtypes 2, 16, 27, 33, 34, 56, 58, 59, 76 in the past [6, 9, 10]. Earlier Kettler et al. suggested that clinical features which can be associated with the presence of HPV in nongenital BD include black race, palmoplantar involvement, verrucous clinical appearance, young age, and history of HPV related genital lesions of different types [11]. Though our patient did not have any of these features, presence of HPV could not be ruled out in the absence of viral examination. HPV infection into host chromosomes leads to downregulation of E6 and E7 proteins indirectly. This promotes malignant change and aids cellular transformation [12]. 

Multiple lesions of BD are often seen in individuals exposed to arsenic. Arsenic exposure toxicity due to several medications including homeopathy has been reported in the past [13, 14]. Our case had no occupational exposure suggestive of arsenic toxicity in the past. Similarly he had no history of chronic arsenicism in his neighbourhood or family. The records of environmental ministry failed to reveal any case of arsenic toxicity or ground water poisoning in the vicinity. It is possible that long term homeopathic medication may be the culprit in our patient. However, this could not be confirmed due to lack of availability of homeopathic medication which he had ingested in the past. Estimation of blood arsenic level was futile because it tends to normalize within a short span of 6 months after nil arsenic exposure [15]. It is known that gradual improvement occurs in signs of chronic arsenicism over a period of 18 months if no further exposure to arsenic occurs [1, 15]. However diffuse pigmentation may remain in such patients [15]. Clinically arsenical BD can be differentiated from nonarsenical BD by its multiple and recrudescent lesions, occurring mainly on sun-protected areas of skin [16, 17]. 

Clinically, in BD, ulceration is a sign of invasive carcinomatous development [1]. 3–10% of untreated BD may progress to invasive carcinoma, usually squamous cell carcinoma [2, 18]. Further 13% of such patients will develop metastasis and 10% will end in death [2]. However presence of pigmentation does not alter the metastatic potential of BD [19, 20]. In an earlier study of multiple BD lesions, 73% of lesions were present on the skin not exposed to sun [15]. 

Differential diagnosis of pigmented BD includes superficial basal cell carcinoma, bowenoid papulosis, seborrhoeic keratoses, pigmented actinic keratoses, melanocytic nevus, blue nevus, and superficial spreading melanoma [1, 2, 21, 22]. Clinically, BD may be differentiated from superficial basal cell epithelioma by the absence of a fine pearly border and lack of a tendency to heal with central atrophy [1, 18]. Pigmented BD usually presents as a nonuniformly pigmented plaque with a scaly or verrucous surface [22]. In a large study, Cameron et al. reported that the most common clinical presentation was a flat or slightly elevated, sharply demarcated, light brown, variegated papule, or plaque with varying degrees of scaling that occurs in men with an average age of 67 years, on the extremities (44%), followed by the trunk [6, 23]. Histopathology is the gold standard for diagnosing and differentiating BD [24]. In BD normal epidermis is replaced by atypical, hyperchromatic, pleomorphic, often large abnormal keratinocytes which show disordered differentiation and loss of epithelial polarity. Epidermis shows acanthosis with elongation of the rete ridges [18]. In some cases, the proliferating cells may be surrounded by relatively normal epidermal cells to give a characteristic “Borst Jadassohn” appearance [1]. Histopathologically, various patterns like psoriasiform, verrucous, pagetoid, hyperkeratotic, atrophic, and pigmented have been mentioned [10]. Numerous vacuolated atypical cells may be seen in arsenical BD [1, 25]. Pagetoid variant of BD is sometimes difficult to distinguish from Paget's disease and from in-situ superficial spreading melanoma [10]. Melanoma cells are positive for S100 proteins, whereas Paget cells usually demonstrate carcinoembryonic antigen [10]. Material in Paget cell is often PAS positive and diastase resistant unlike BD where it is PAS positive and diastase labile [18]. 

Pigmented BD is characterized by increased melanin pigment in basal cells of epidermis, melanocytes and dermal melanophages [20]. Exact mechanism of pigmentation in BD is not yet known. It has been suggested that neoplastic cells may produce specific factors or cytokines that induce proliferation of melanocytes and stimulate melanin synthesis [20]. Satter proposed that the pigmentation is due to the presence of an increased number of enlarged melanocytes with hypertrophic dendritic processes dispersed throughout the neoplasm [26]. It has been argued that incidence of pigmented BD has been underreported in the past [27]. Due to high sensitivity of dermoscopy in detecting pigmentory changes, newer studies have reported a much higher incidence of pigmented variant of BD, to the tune of 38–64% [28]. Glomerular vessels, scaly surface, small brown globules, reticular pigmentation, and homogenous pigmentation are the dermoscopical features of BD [24, 28]. Disappearance of glomerular vessels, small brown globules, and reticular pigmentation may be useful dermoscopic features in the followup of pigmented BD after therapy [24]. In the past, most of the pigmented variants of BD were reported to affect intertriginous areas [10, 21]. However, at present the majority of them are regarded as Bowenoid Papulosis [10]. 

BD has a high recurrence rate of 10–15% [6, 10]. In the absence of a clear cut superior treatment option, multiple therapeutic modalities have been tried for BD. Curettage with excision and/or cryotherapy is the treatment of choice for a solitary BD [6, 29]. Topical 5-fluorouracil, photodynamic therapy, laser ablation, topical imiquimod, and radiotherapy are the other modalities worth trying [6]. However multiple BD is a therapeutic challenge with a tendency to recur [6, 29]. For multiple lesions, guidelines given elsewhere state cryotherapy as a good choice followed by curettage, photodynamic therapy, and topical 5-fluorouracil as a fair choice [6]. While topical imiquimod, radiotherapy, and lasers have been clubbed as reasonable choices, excision is a relatively poor choice in patients with multiple BD. However many of these modalities are inaccessible due to the cost or availability of specialized equipments. Earlier report has used acitretin to treat multiple arsenical keratoses and BD [10]. As our patient had tried most of these modalities he was counselled and started on NBUVB (narrowband UVB) after informed written consent. Baseline biopsies were taken from multiple plaques for ascertaining the response of phototherapy. Unfortunately he was lost to follow-up after three weeks. During this period there was no perceptible clinical change in his lesions. 

Though sunlight exposure is implicated as a cause of BD, most of the lesions of arsenical BD occurs more often on the sun-protected skin [16]. Why is it so? Does this mean that sun exposure has a therapeutic role in arsenical BD? This fact has not been investigated much in past studies. Lee et al. reported that combination of UVB and arsenic resulted in antiproliferative and proapoptotic effects on the keratinocytes. Further they postulated the potential therapeutic role of UVB in arsenic induced BD [30]. This may have long term ramifications for treatment of arsenical BD. However this hypothesis is open to argument in our patient, for the lack of viral, mutational, and molecular analyses and further studies are required to substantiate this claim. 

## 4. Conclusion 

To conclude, pigmented BD should be considered as a differential diagnosis of any long-standing pigmented skin lesion. Possibility of a cutaneous malignancy, however remote, should always be considered and ruled out by a skin biopsy [31]. It may be difficult to ascertain the etiology in patients with multiple BD. In patients with multiple lesions of BD, treatment related morbidity and ease and availability of treatment options are a greater issue than actual cure [6]. Though the available literature indicates that serum arsenic level returns back to normal after a period of nil arsenic exposure, its noteworthy that the lesion of BD may progress with time even if there is no further exposure to arsenic. This may have some implications on the etiopathogenesis of BD. Our case is noteworthy for its unusual and widespread clinical presentation. Multiple BD is a therapeutic dilemma and nightmare for treating dermatologist. In paucity of clearcut guidelines for multiple BD, the present case raises a valid argument for trying UVB therapy as a therapeutic modality in patients of multiple arsenical BD. Besides, generalised expression of pigmented lesions in BD is hitherto unreported in type V Fitzpatrick skin.
